# Morphological and Gene Expression Changes in Cattle Embryos from Hatched Blastocyst to Early Gastrulation Stages after Transfer of In Vitro Produced Embryos

**DOI:** 10.1371/journal.pone.0129787

**Published:** 2015-06-15

**Authors:** Jessica van Leeuwen, Debra K. Berg, Peter L. Pfeffer

**Affiliations:** 1 AgResearch Ruakura, Animal Productivity Section, Hamilton, New Zealand; 2 Department of Biological Sciences, University of Waikato, Hamilton, New Zealand; 3 School of Biological Sciences, Victoria University of Wellington, Wellington, New Zealand; Laboratoire de Biologie du Développement de Villefranche-sur-Mer, FRANCE

## Abstract

A detailed morphological staging system for cattle embryos at stages following blastocyst hatching and preceding gastrulation is presented here together with spatiotemporal mapping of gene expression for *BMP4*, *BRACHYURY*, *CERBERUS1 (CER1)*, *CRIPTO*, *EOMESODERMIN*, *FURIN* and *NODAL*. Five stages are defined based on distinct developmental events. The first of these is the differentiation of the visceral hypoblast underlying the epiblast, from the parietal hypoblast underlying the mural trophoblast. The second concerns the formation of an asymmetrically positioned, morphologically recognisable region within the visceral hypoblast that is marked by the presence of *CER1* and absence of *BMP4* expression. We have termed this the anterior visceral hypoblast or AVH. Intra-epiblast cavity formation and the disappearance of the polar trophoblast overlying the epiblast (Rauber’s layer) have been mapped in relation to AVH formation. The third chronological event involves the transition of the epiblast into the embryonic ectoderm with concomitant onset of posterior *NODAL*, *EOMES* and *BRACHYURY* expression. Lastly, gastrulation commences as the posterior medial embryonic ectoderm layer thickens to form the primitive streak and cells ingress between the embryonic ectoderm and hypoblast. At this stage a novel domain of *CER1* expression is seen whereas the AVH disappears. Comparison with the mouse reveals that while gene expression patterns at the onset of gastrulation are well conserved, asymmetry establishment, which relies on extraembryonic tissues such as the hypoblast and trophoblast, has diverged in terms of both gene expression and morphology.

## Introduction

There are two main reasons why it is important to better understand the early developmental events in cattle. First, cattle are commercially important for dairy as well as meat production. Herd maintenance and in particular lactation is reliant on efficient reproduction. However, it is known that the greatest gestational losses, namely 28% in beef and moderately producing dairy cows and up to 40% in high producing dairy cows, occur within the first three weeks of fertilisation [1, 2]. In particular, the bulk of losses are seen in the second week of gestation [3–8], during which the bovine blastocyst embryo hatches from the surrounding proteinaceous zona pellucida shell and develops its initial three lineages, the embryonic epiblast and the extraembryonic hypoblast and trophoblast [9]. A recent transgenic model has indicated that the epiblast and hypoblast lineages are particularly sensitive to perturbations [10], which suggests that defects in the development of these lineages may cause the high embryo losses seen. Little is known as to the morphogenetic and molecular events leading to the patterning of the epiblast and indeed whether the underlying hypoblast shows any patterning at all. Such knowledge is required to diagnose, understand and potentially alleviate the declining fertility seen in dairy cattle.

Secondly, most of our understanding of mammalian embryology comes from studies on the mouse. However, mice display some embryological features, such as a cup-shaped epiblast [11], the maintenance of the polar trophoblast [12], early epiblast cavitation that leads to the amniotic cavity, precocious allantois formation [13] and a complex set of specialised cells involved in invasive implantation [14], that are non-typical for eutherian mammals and even for other rodents. With a rapid life cycle of 9 weeks (3 weeks gestation and 6 weeks postnatal to maturity), mice have been subject to many more generations (rounds) of natural selection than larger mammals with generation times measured in years (cattle: 2.5 years/130 weeks; humans: >12 years) since sharing a common mammalian ancestor. Mice are therefore likely to have diversified more from the ancestral state than their cousins. Hence the study of alternate mammals should be enlightening in terms of understanding features that are of ancestral mammalian origin. While progress has been made recently in establishing rabbits [13, 15–17] and pigs [18–20] as embryological model systems, cattle are less well characterised [21, 22] yet are of high interest in that they represent a large suborder of mammals, namely the ruminants, consisting of 250 distinct species, many of which are of economic importance to humans.

In cattle, as in all eutherian mammals examined so far, fertilisation is followed by a series of cleavage divisions leading to a blastocyst consisting of an outer layer of trophoblast cells encapsulating a mass of cells (the ICM) apposing the TE on the “embryonic” pole with the blastcoel cavity filling the rest of the internal space. After E7, the ICM further differentiates into the epiblast and hypoblast. The hypoblast (sometimes called the primitive endoderm) forms a layer lining the blastocyst cavity concomitant with hatching out of the zona pellucida at E9-10 [21]. The hypoblast has a role not only in early nutrient exchange but is required for anterior-posterior patterning of the epiblast-derived embryo proper [23] We here describe the further development of these first three lineages until the start of gastrulation. Expression was analysed for genes whose homologues mark distinct tissues in the early the mouse embryo. These were the polar trophoblast markers *Furin* [24], *Bmp4* [25] and *Eomesodermin* (*Eomes*) [26], the epiblast markers *Nodal* [27] and *Cripto (Tdgf1*) [28], the anterior hypoblast markers *Cerberus1* (*Cer*1) [29] and the prospective mesoderm markers *Brachyury* (*T*) [30] and *Eomes* [31]. In mice, *Cer1* is additionally expressed in anterior mesendoderm cells and their progenitors in the primitive streak [29, 32]. We specifically chose these seven marker genes as they have all been functionally implicated in the patterning of the mouse embryo and thus were deemed most informative for understanding the mechanisms driving early development in cattle.

## Materials and Methods

### Embryo generation

All animal work carried out was specifically approved by the Ruakura Animal Ethics committee RAEC 12025 (Hamilton, New Zealand) and all efforts were made to minimise suffering. Ovaries were obtained from local abattoirs of predominately dairy cow origin. In vitro produced (IVP) Embryos were generated as described previously [4]. Day zero was taken as the day of fertilisation. Frozen-thawed semen from a single Friesian bull was used for in vitro fertilisation (IVF). On day seven following IVF, embryos were morphologically graded and grade one and two blastocysts were selected for transfer. Embryos were washed and held in EmCare hold (ICPbio, Auckland, NZ) before trans-cervical insertion into the ipsilateral uterine horn of synchronised recipient animals. Eight to 15 embryos were transferred to each recipient animal. Recipient animals were maintained under pastoral conditions and were either parous non-lactating dairy cows or 15–18 month-old crossbred beef heifers. Recipient animals were synchronised using a single intra-vaginal progesterone releasing device for 10 to 12 days (CIDR-b; InterAg, Hamilton, NZ). Four days before the CIDR was removed cattle received 500 μg of cloprostenol (Estroplan; Parnell Laboratories, Auckland, NZ). Cattle were checked for oestrus three times daily and only those which had displayed oestrus and had a palpable corpus luteum were used as recipients.

Embryos were recovered at gestational age 11–15 post IVF via post slaughter flushing of the uterine tract or nonsurgical flushing using an embryo collection catheter [4]. EmCare hold was used for flushing and for searching/holding the embryos in. Embryos to be used for real time PCR analysis were microdissected to separate the embryonic disc using tungsten needles or ultra sharp splitting blades (Bioniche Animal Health, USA). Embryonic discs and trophoblast tissue from each embryo were washed briefly in PBS before separately being homogenised in 100 μl of trizol (Invitrogen life technologies, Auckland, NZ) and snap frozen on dry ice. They were then stored at -80°C. Embryos to be used for in situ hybridisation were fixed in 4% paraformaldehyde (Sigma)/PBS for 4–6 hours on ice before being dehydrated through methanol steps and stored at -20°C in 100% methanol until use.

### Probe preparation

The NCBI RNA reference sequence for cattle of selected genes was used to design primers (5’ to 3’; forward and reverse):

*CERBERUS1* (XM_584735), AGCTGCTGGTGCTCCTGCCT and CCTGTGCGGGGTAGCCATGC;

*BRACHYURY* (XM_864890), GCTTCACAAGGAGCTCACCAAC and AAGGCTGGACCAGTTGTCAT;

*CRIPTO* (NM_001080358), GCTTTCCTCAGTCATTCCT and AACAGGTGCCCTTGTCTCAT;

*FURIN* (NM_174136.2), CATCTACACGCTGTCCATCA and CCATAAAGCACGAGGGTGA;

*NODAL* (XM_609225), GCAGGTGGATGGGCAGAACT and CATTCCTCCACAATCATGTC;

*EOMESODERMIN* (XM_001251929), CTTCAGGGACAACTATGATT and CGCTTACAAGCACTGGTGTATA;

*BMP4* (NM_001045877), CTCAGGGCAGAGCCATGAGCT and CGTTCTCTGGGATGCTGCTGA.

A 50 μl PCR reaction was run using Roche Fast start Taq polymerase following the manufacturer’s instructions (Roche, Auckland, NZ) with an annealing temperature of 60°C. Template cDNA kindly supplied by Dr Craig Smith was from a bovine day 14 embryo and for *BRACHYURY* a day 16 embryonic disc. The gel and Wizard (Promega, Auckland, NZ) purified DNA product was inserted into the pGEM-T-Easy vector (Promega) following the manufacturer’s instructions and sequence verified. Depending on insert orientation either T7 or SP6 RNA polymerase (Roche) were used with the DIG RNA labelling mix (Roche) to generate sense and antisense RNA probes. Probes were purified using RNA-quick spin columns (Roche).

### Whole mount in situ hybridisation (WMISH)

The WMISH protocol used was based on the protocol by [33] with buffer details (HB, MABT, NTMT) listed therein. Steps were carried out in 2 ml round bottomed microfuge tubes with gentle rocking (24 rpm) at room temperature (RT) with washes being 5 min unless specified differently. Embryos stored in 100% methanol were treated with 3% hydrogen peroxide in methanol for 1 hour at room temperature. They were then rehydrated through 75%, 50%, 25% methanol/PBS for 10 min for each step. Larger embryos were cut to remove excess trophectoderm tissue. Embryos were washed twice in PBT (PBS with 0.1% Tween-20, Sigma-Aldrich), digested with 10 μg/ml proteinase K (Roche) in PBT for 8–10 min depending on size. Embryos were then washed in 2 mg/ml glycine in PBT followed rapidly by 2 rinses in PBT. Embryos were post fixed for 20 min in 4% paraformaldehyde/0.1% glutaraldehyde/PBT, washed twice in PBT and once in 50% hybridisation buffer (HB)/50% PBT. This was replaced with HB and the embryos were rocked at 65°C for at least one hour, then with 1 ml 65°C HB containing 5% dextran sulphate (Sigma-Aldrich D6001) and 1 μg/ml of DIG labelled RNA probe. Embryos were rocked overnight at 65°C, then washed 2 x 30 min at 65°C in HB, once at 65°C with 50% HB/50% MABT, twice with MABT, twice MABT-500 and treated for 1 hour with 10 μg/ml RNAse A/MABT-500 at RT, washed twice in MABT-500 and MABT and blocked for an hour each in MABT with 10% Boehringer blocking reagent (BBR, Roche, 1096176001) and 10% BBR/10% heat treated lamb serum/MABT. Embryos were rocked overnight at 4°C in MABT/10%BBR/10% lamb serum and 1/2000 dilution of anti-DIG-Alkaline phosphate FAB fragments (Roche 11093274910), then washed twice with MABT, transferred to 20 ml glass scintillation vials and washed 3 x 1 h with 20 ml MABT. Embryos were sliced open to ensure stain would not get trapped and transferred to airtight 5ml glass scintillation vials and washed twice for 10 min with NTMT. This was replaced with 3 ml of NTMT containing 0.23 mg/ml NBT (Roche, 11383213001) and 0.11 mg/ml BCIP (Roche, 1383221001) that was syringe filtered through a MILLEX-HA 45 μm syringe filter (Millipore, SLH033SS). Embryos were rocked in the dark for up to 3 days, then rinsed in PBT and photographed using a glass concavity slide using the Leica AF6000 system (Leica DMI6000B microscope, DFC300FX camera and Leica application suite software version 2.5.0). Number of embryos subjected to WMISH (and sectioned) were: *CRIPTO* 7 (2), *FURIN* 8 (4), *NODAL* 16 (13), *EOMES* 5 (4), *CER1* 16 (10), *BMP4* 7 (2), *BRACHYURY* 7 (4).

### Histology

Embryos to be sectioned were embedded in 4% agarose (Fisher Biotech, Wembley, Australia) and cut into trapeze shapes for orientation. The embryo blocks were then manually dehydrated through a 25%, 50% and 70% ethanol series for 10 minutes each before processing to histowax (Histoplast PE, Thermoscientific) in a Leica TP1050 tissue processor. The processor steps were as follows and were all for 1 hour at 40°C and ambient pressure unless specified: twice 70% ethanol at RT, twice 95% ethanol, thrice absolute ethanol, once each 50% ethanol/50% xylene 80 min, xylene 45 min, xylene 45 min under vacuum, thrice in histowax for 80 min at 60°C under vacuum. The wax-infiltrated agarose blocks were embedded into paraffin blocks using a Thermolyne Histo-Center II-N and sectioned at 7 μm using a Reichert Jung microtome 2050 Supercut. Sections were mounted on polylysine coated slides (Labserv, Auckland, NZ) or stained with Haematoxylin and Eosine before mounting in DPX (Sigma Aldrich).

Some slides were stained with phalloidin-conjugated tetramethylrhodamine isothicyanate (TRITC; Sigma P1951) to visualise actin and counterstained with DAPI to view cell nuclei. Slides were soaked in xylene for 3 days to remove the coverslips and then immersed in two changes of fresh xylene for 10 minutes each. They were then rehydrated through an ethanol series, washed twice with PBS, permeabilised for 5 minutes with 0.1% Triton X-100 (Sigma Aldrich) in PBS, and blocked in PBS containing 1% (w/v) BSA for 1 hour at room temperature. Slides were incubated with 5 μg/ml phalloidin-TRITC for 1 hour at room temperature before washing three times with PBS. They were then incubated for 20 minutes at room temperature with 0.75 μg/ml DAPI in PBS, washed with PBS and mounted in fluorescent mounting medium (Dako, Carpinteria, CA, USA S3023) and visualised under fluorescent light.

### RT-PCR

Total RNA isolation, DNAse treatment and reverse transcription was carried out as described [34] with the exception that the final step through a column was replaced with an ethanol precipitation. For detection, SYBR-green real time PCR was used using a Corbett RG-6000 instrument. Each 10 μl reaction contained 5 μl Takara SYBR premix Ex Taq (Takara), 1 pmol of each primer, 2 μl of cDNA and 2.8 μl of super clean water. The thermal cycling programme included a 3 min incubation at 95°C to activate the enzyme followed by 40 cycles of 95°C for 10 sec, and annealing/extension at 60°C for 35 sec. Green fluorescence was measured during the last 20 sec of the anneal/extension phase. The cycles were followed by a melt step from 72 to 99°C. All primer pairs were designed to give a product of 200–300 bp. The melt curve was checked to ensure only one product was produced and this was also run on an agarose gel to check the expected product size was produced. Each RT-PCR run included a no template control and an RT- control. Samples were measured in triplicate with one measurement being a two-fold dilution to ensure measurement was occurring during the linear phase of amplification. A relative copy number for each transcript was calculated using a variation to the 2^-ΔΔCt^ method [35] and normalising this to the geometric mean of three housekeeping genes (*GAPDH*, *CYCLOPHILLIN* and *HPRT*). In the 2^-ΔΔCt^ calculation the ‘2’ was replaced with the actual reaction efficiency as calculated by the Corbett software and the ‘C_t_’ value used was the ‘take-off cycle’ which is when the reaction is at 20% of the maximum level, and indicates the end of the noise and the transition into the exponential phase. Primers were as follows, 5’ to 3’, *CERBERUS1*, AGGACAGTGCCCTTCAGCCA and CCTGTGCGGGGTAGCCATGC; *EOMES*, CTCCCATGGACCTCCCGAACAA and AGACAGCCGCCTYCGCTTACAA; *FURIN*, AGATGGGTTTAACGACTGGG and CCATAAAGCACGAGGGTGA and the housekeeping genes *GAPDH*, *CYCLOPHILLIN* and *HPRT* as in [36].

## Results

### Morphological events in relation to epiblast size

**Fig 1:**
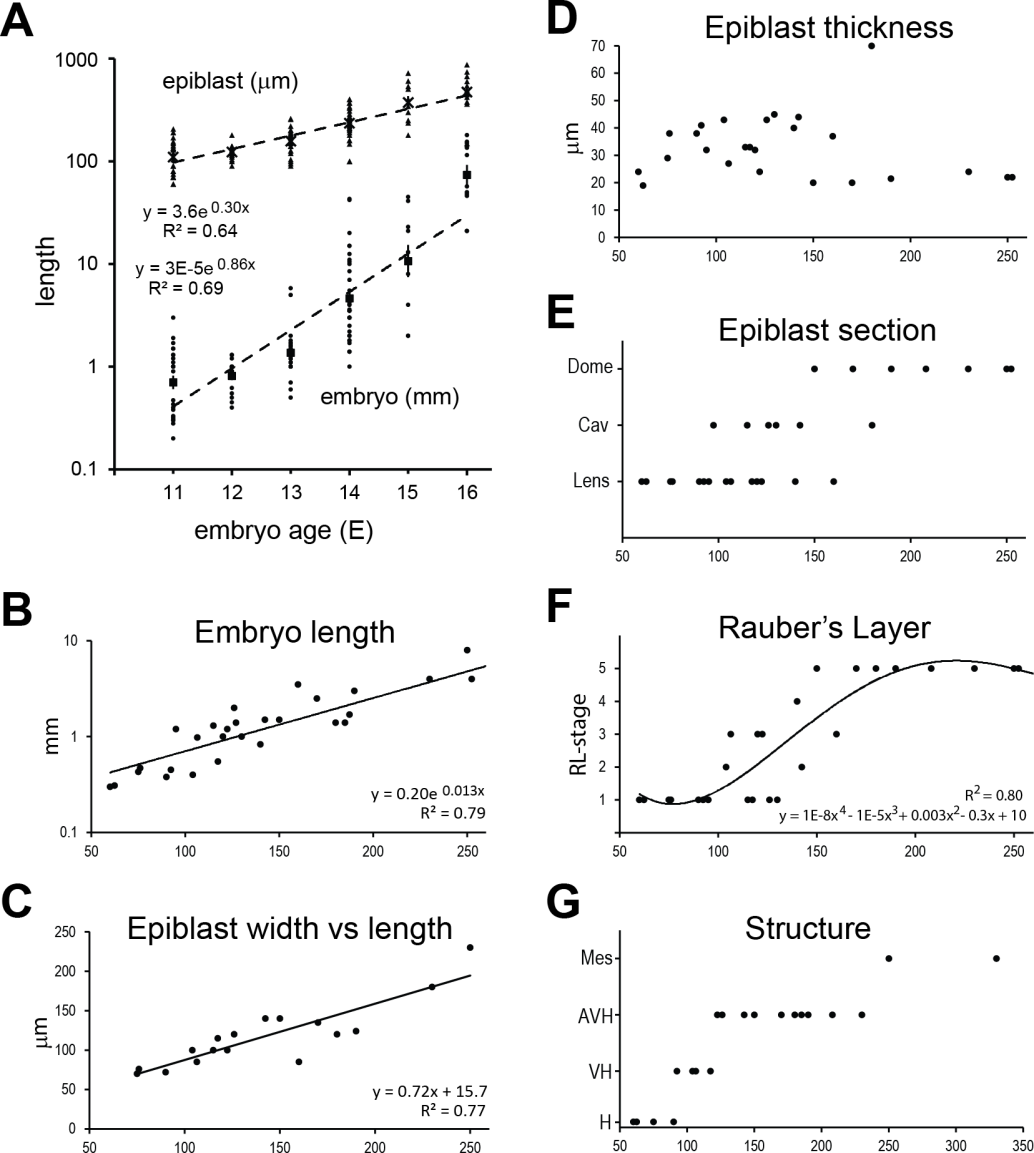
Quantification of morphological traits relative to embryonic age or epiblast size. A. Embryo length in mm (triangles; crosses geometric mean) and epiblast length in μm (dots; solid squares geo-mean) are plotted on a log scale against embryo age; n = 126. B-G. Morphological traits plotted against the maximal diameter of the epiblast (length) in μm; n = 32. In E., the shape of the epiblast in transverse section has been categorised as “Lens” for concave lens shaped epiblast without cavities, as “Cav” for concave epiblasts with intra-epiblast cavities and as “Dome” for embryonic ectoderm (EmE) discs protruding above the surface of the embryo, predominantly 2-cell layers thick. F. Rauber’s Layer (RL)-stage has been assigned as per Table 1. G. “Structure” refers to the additional embryonic structure that becomes visible at the indicated epiblast size: H, undifferentiated hypoblast; VH, both VH and PH are visible; AVH; differentiation of VH into AVH is visible; Mes, in addition to the AVH, endomesodermal cells are seen.

Whereas for foetal (postembryonic) stages a chronological classification scheme for developmental stage may be adequate, this is not the case for early development. The exact chronological age of retrieved embryos in this study is known by virtue of transferring in vitro grown blastocyst embryos into hormonally synchronised recipients. Fig 1A indicates that while there is a high correlation of embryonic age with two of the measures of embryonic stage, namely embryo (trophoblast) size and epiblast length, there is a huge range of sizes at any given day of embryo age.

**Fig 2:**
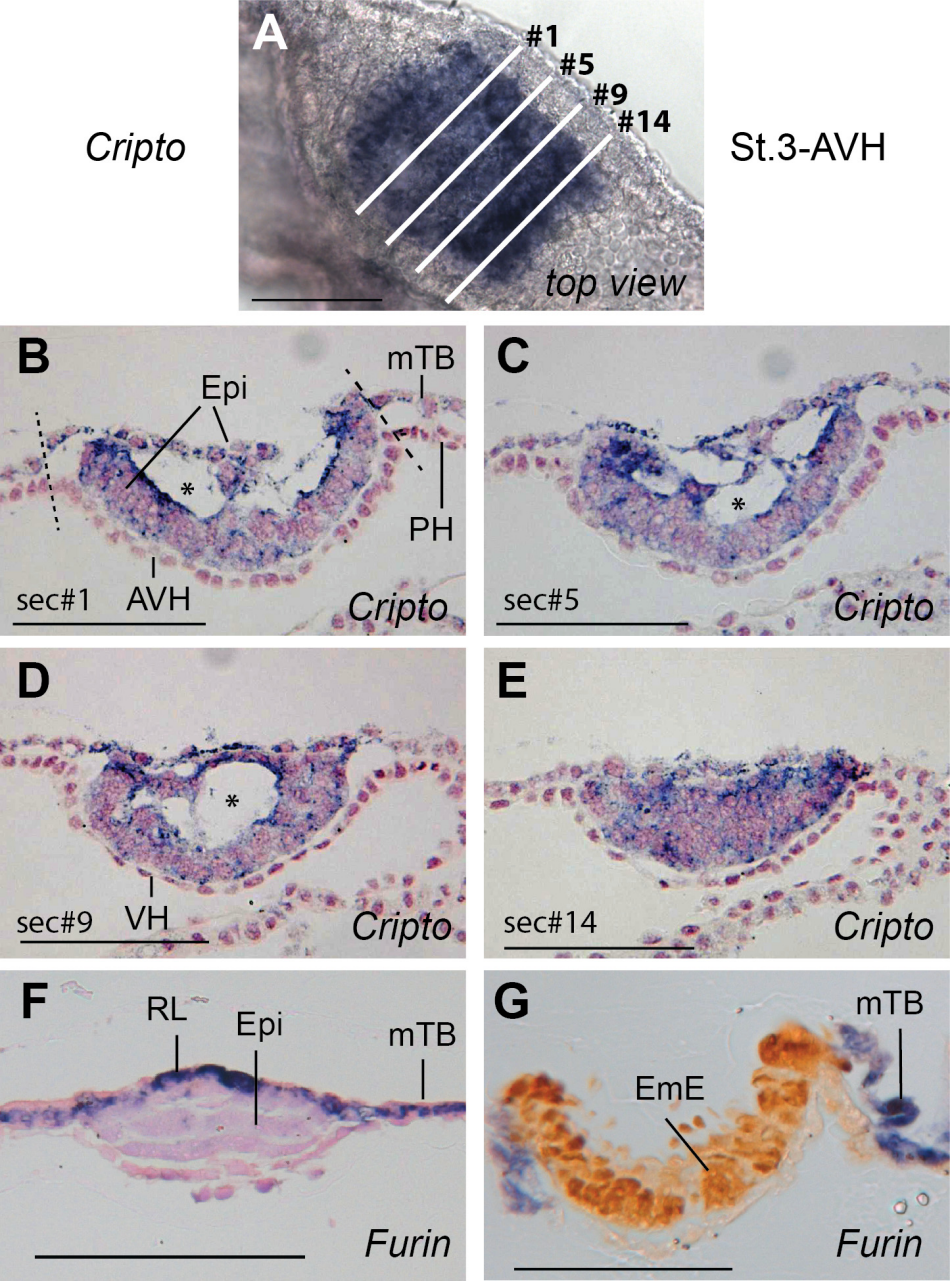
Intra-epiblast cavitation and RL disappearance as marked by *CRIPTO* and *FURIN* expression respectively. A. *CRIPTO* expression in a stage 3-AVH embryo with a 160 μm epiblast before sectioning. B-E. Progressive cross-sections of embryo shown in panel A. Section numbers are indicated, each section was 8 μm thick. The progressive merging and eventual rupturing of large cavities within the epiblast can be seen in this series. No RL remained in this embryo as seen by *CRIPTO* expression throughout the disc. The inner region of the epiblast has already assumed an EmE-like 2-cell layer appearance in panels B-D. Star, intra-epiblast cavity. F, G. *FURIN* expression in F., stage 2-VH and G., stage 4-EmE embryos, showing expression in RL as well as the mural trophoblast (mTB). Bars represent 100 μm.

**Fig 3:**
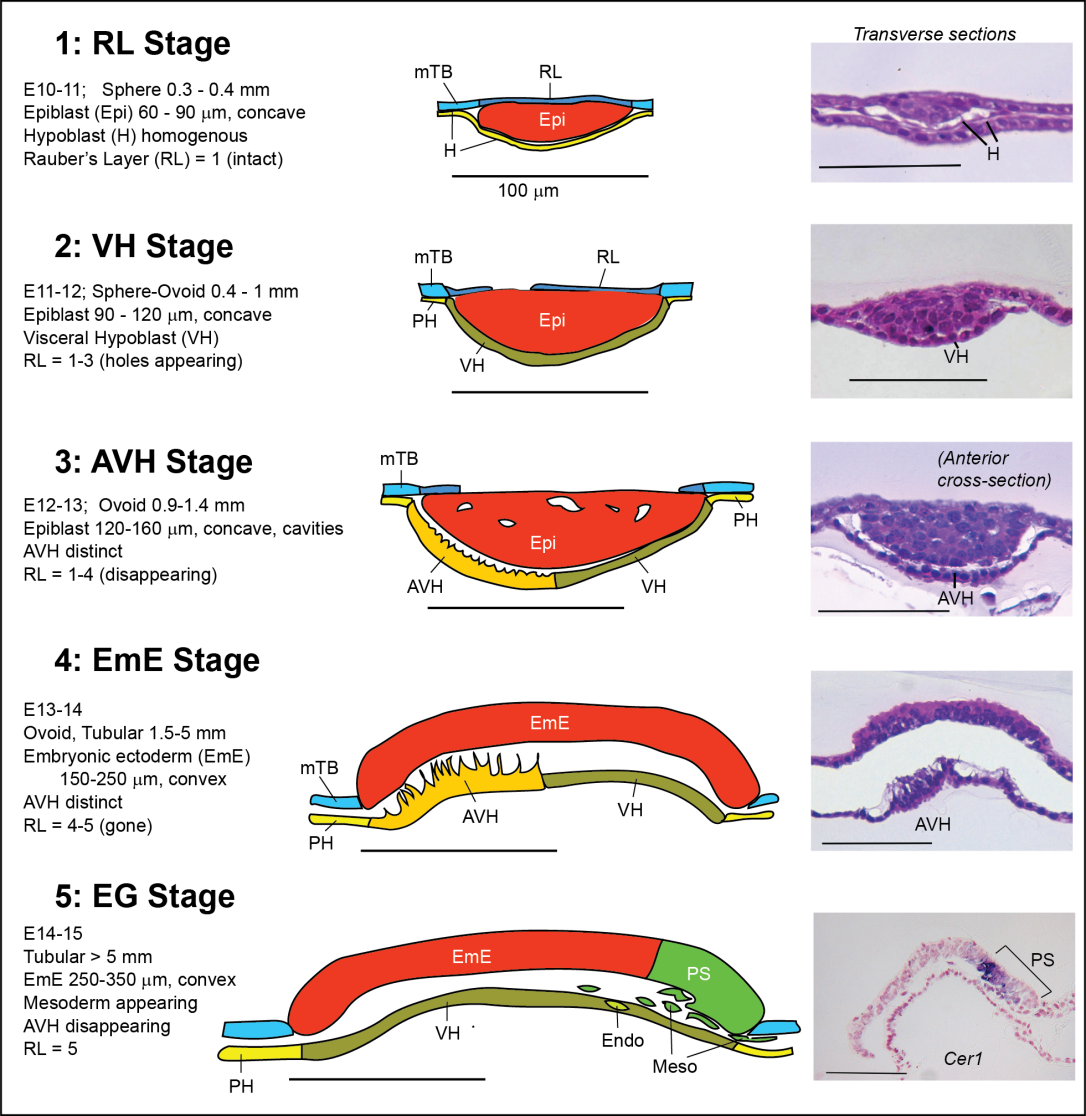
Cattle embryo staging system from post-hatching to the start of gastrulation. Criteria for, and diagrammatic representation and typical sections (H&E or *CER1* stained) of, cattle embryos at the five stages of development between hatching and the start of gastrulation, based on data from sectioning 32 embryos. All bars are 100 μm. AVH, anterior VH; EmE, embryonic ectoderm; Endo, endoderm; Epi, epiblast; H, undifferentiated hypoblast; Meso, mesoderm; mTB, mural trophoblast; PH, parietal (mural) hypoblast; PS, primitive streak; RL, Rauber’s Layer (polar TB); VH, (embryonic) visceral hypoblast.

**Fig 4:**
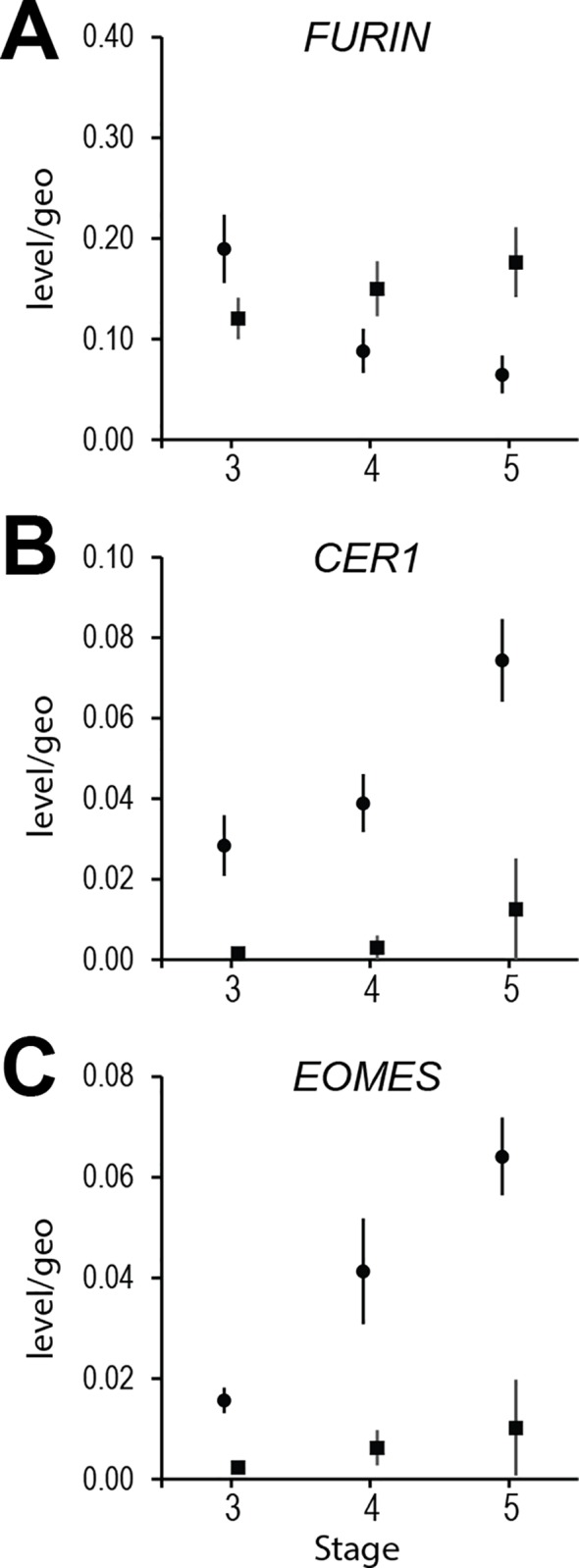
Gene expression via real time PCR. A. *FURIN*, B. *CER1* and C. *EOMES* expression relative to the geomean of three housekeeping genes (see Methods and S3 Fig) in 3-AVH to 5-EG stage micro-dissected embryos. Solid circles represent embryonic discs (with remaining RL material at stage 3-AVH) whereas squares are mural TB. Error bars are s.e.m.. Sample sizes as follows: for discs, stages 3, 4, 5; *n* = 7, 9, 15; for mTB, stages 3, 4, 5; n = 4, 8, 7; where some of the *n* samples were themselves pools of two embryos.

**Fig 5:**
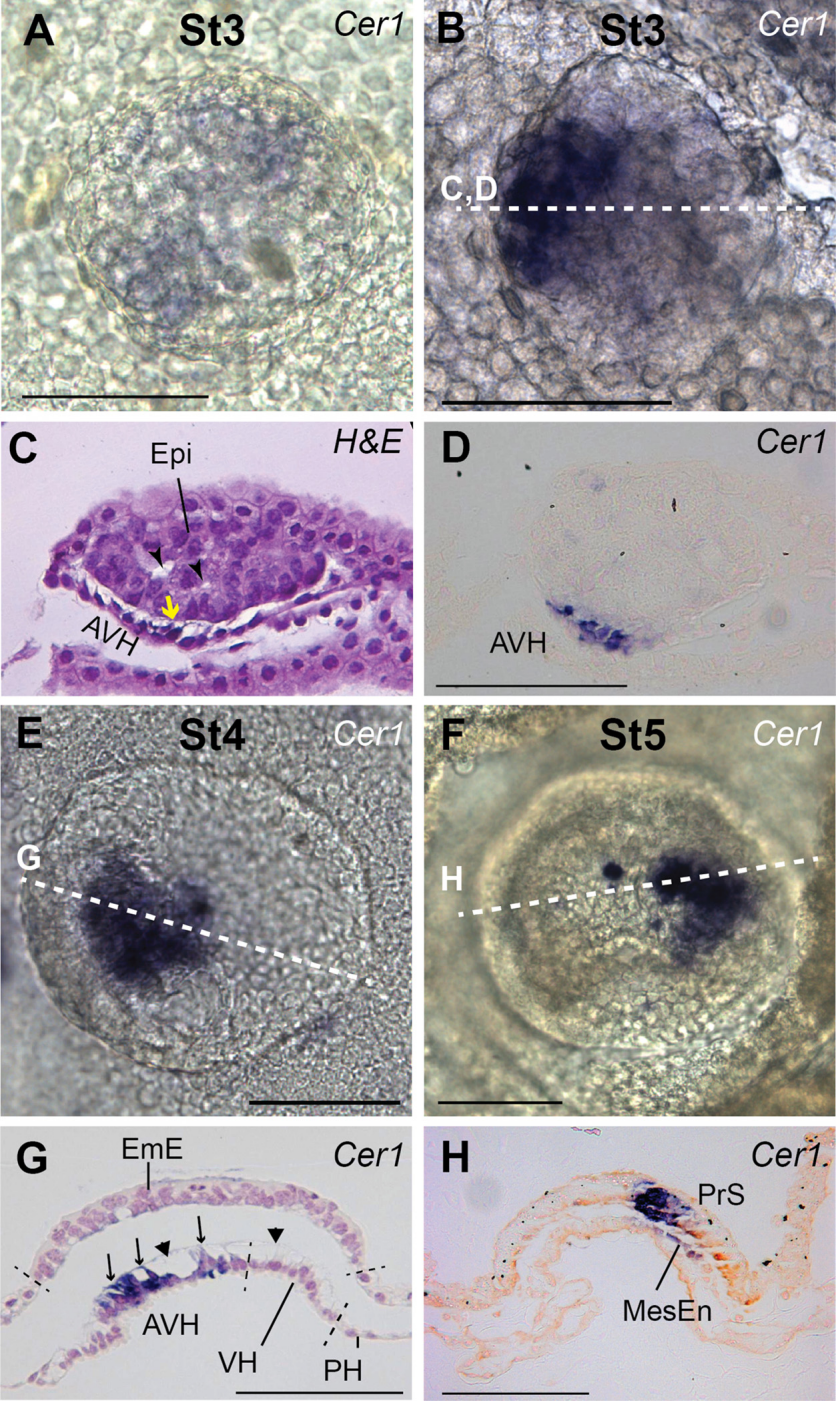
*CER1* expression. All embryos are orientated with anterior to the left, with dorsal (top) views (A, B, E, F) and representative cross-sections as indicated by stippled lines. A. Asymmetric onset of *CER1* at stage 3-AVH. B. Slightly later stage 3-AVH embryo. C. H&E stained section of embryo B showing distinct AVH cells of increased height and with extensions toward epiblast (yellow arrow). Intra-epiblast vacuoles can be seen (arrowheads). D. Section of embryo B adjacent to section C (7 μm) showing confinement of *CER1* staining to AVH cells. E., G. Stage 4-EmE embryo with *CER1* marking the AVH, which still exhibits extensions toward EmE (arrows). The entire visceral hypoblast contains vacuoles (arrowheads) covered by a membrane on the EmE side. F, H. Stage 5-EG embryo without anterior hypoblast *CER1* staining but showing *CER1* expression in the anterior part of the primitive streak as well as in delaminating cells which are presumptive mesendoderm cells (MesEn). Bars, 100 μm.

**Fig 6:**
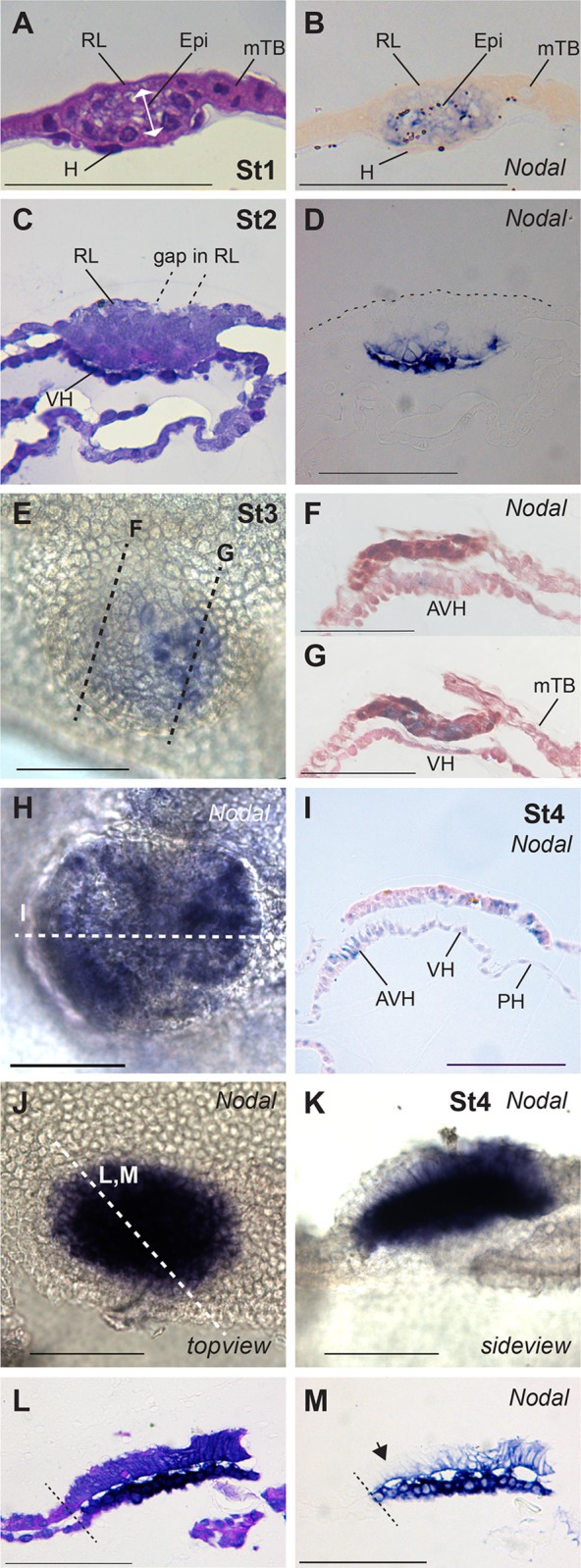
*NODAL* expression. A, B. Adjacent sections of stage 1-RL embryo after WMISH for *NODAL*, stained with H&E (A) or not (B). *NODAL* is restricted to the epiblast and not expressed in the hypoblast underlying the epiblast, which is indistinguishable from that underlying the mural TB. C, D. Adjacent H&E and Nodal sections of stage 2-VH embryo. Only the visceral hypoblast (VH) and inner (ventral) epiblast are *NODAL*-positive. Rauber’s layer (RL) is starting to disintegrate. E, F, G. By stage 3-AVH, *NODAL* expression levels are weaker. *NODAL* is seen in the AVH (section F) and in the epiblast is confined to posterior region (section G). H, I. By stage 4-EmE, *NODAL* is still restricted to the AVH in the hypoblast tissue and is expressed only in the posterior EmE. J-M. Overstained stage 4-EmE embryo indicating expression throughout VH but only presumptive posterior EmE expression (arrow in M., anterior EmE). Bar, 100 μm.

**Fig 7:**
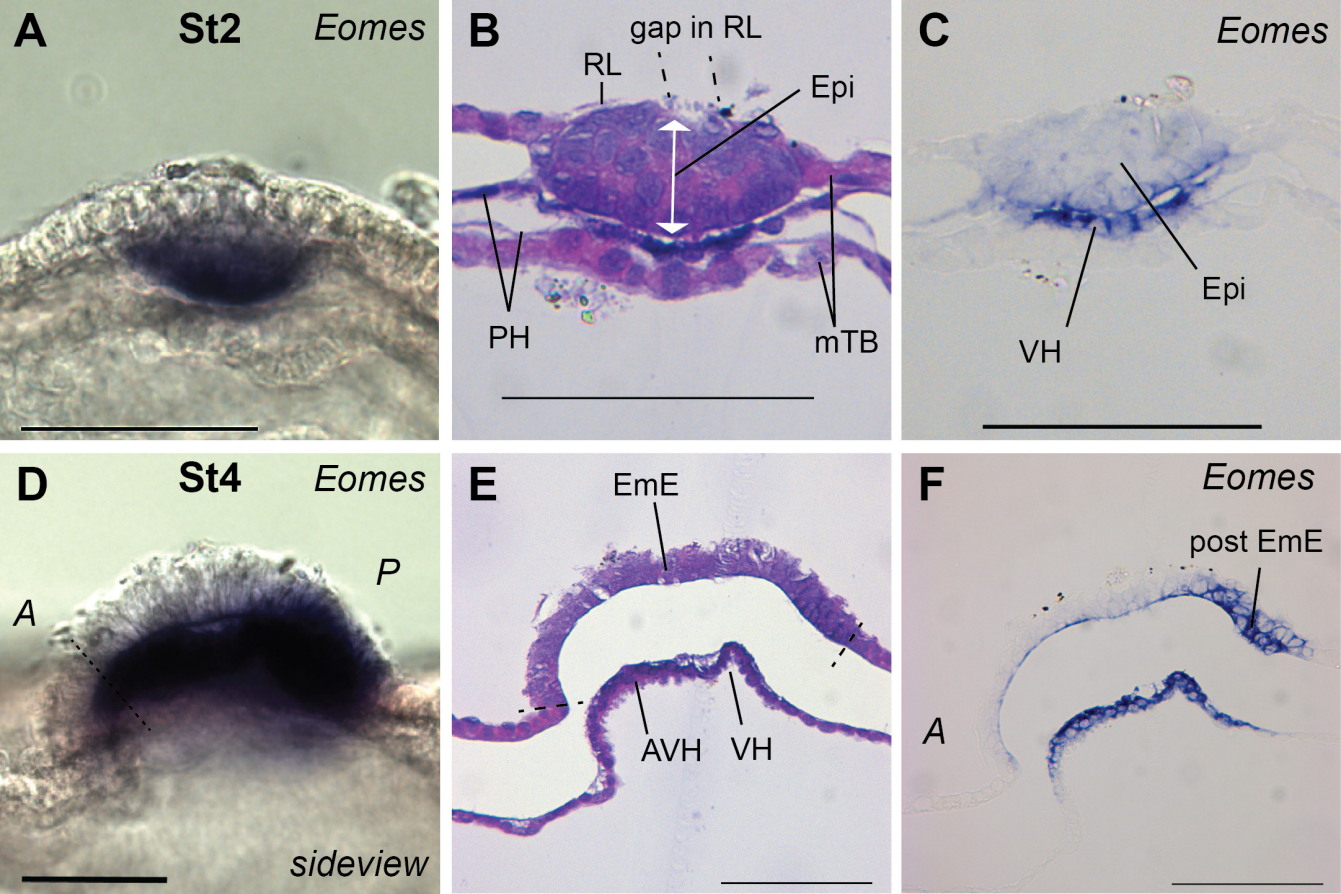
*EOMES* expression. *EOMES* WMISH of stage 2-VH (A, sideview; B, H&E stained section; C, adjacent section) and advanced stage 4-EmE (D-F) embryos. At both stages the visceral hypoblast (VH) is stained. D-F. At stage 4-EmE, the posterior EmE, which will form the primitive streak, is *EOMES*-positive. E and F are adjacent mid-saggital sections, anterior to the left. Bar, 100 μm.

**Fig 8:**
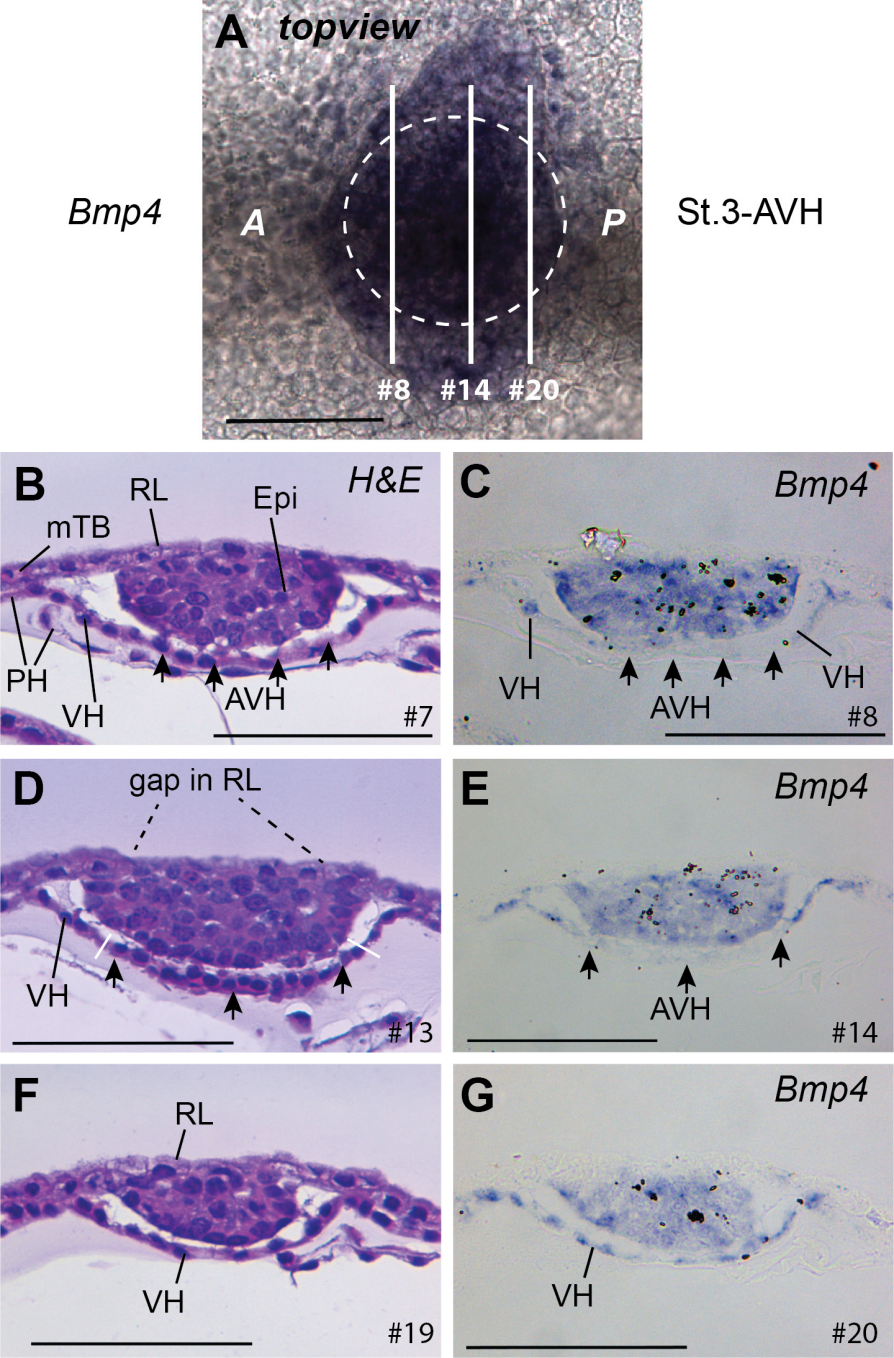
*BMP4* expression is excluded from the AVH. A. Stage 3-AVH embryo showing BMP4 staining extending beyond the embryonic disc which is shown by a stippled circle. B-G. Pairs of adjacent sections as indicated in panel A. The # numbers refer to section, each being 7 μm thick. The AVH is shown via arrows and can be seen to be excluded from *BMP4* stain, which extends into the parietal hypoblast (PH) as well as labelling all the epiblast. Bar, 100 μm.

**Fig 9:**
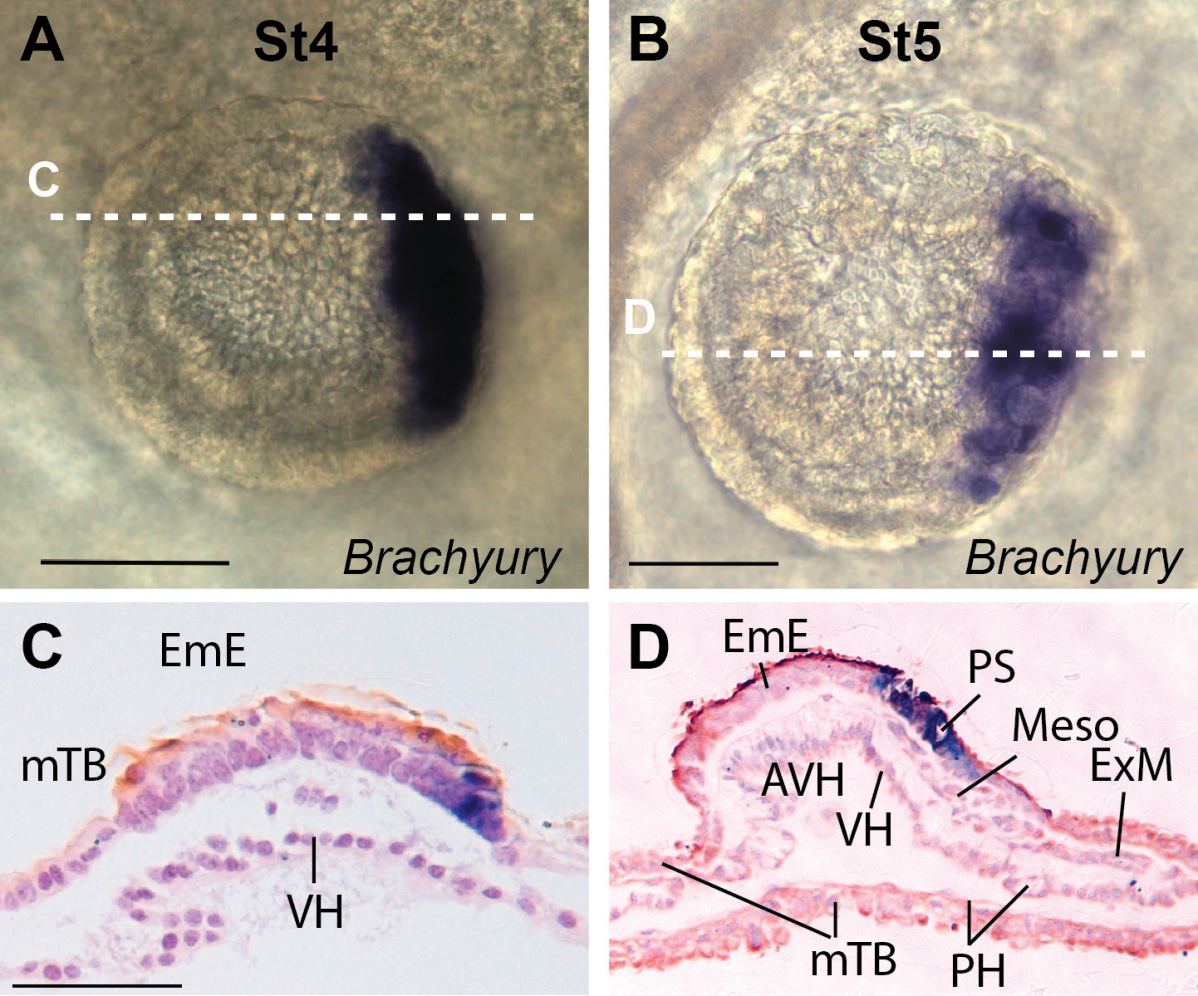
*BRACHYURY* expression commences at stage 4-EmE. A, B. Stage 4-EmE and 5-EmE whole mount embryos stained for *BRACHYURY*. C, D. Saggital sections of embryos A and B, respectively, anterior to the right. D. *BRACHYURY* is strongly expressed in the primitive streak (PrS) but only weakly in the nascent Mesendoderm cells (Meso). Extraembryonic mesoderm (ExM) can be seen separating the mTB and PH posterior to the embryo. Bars, 100 μm.

As the ICM undergoes the most morphogenetic and thus measurable changes before gastrulation, we based our staging system initially on a continuous variable of its lineage, namely the length of the epiblast. When comparing epiblast length to the log of embryo length, a very high correlation of R = 0.9 (R^2^ = 0.79) was seen (Fig 1B). We next examined epiblast characteristics. The dorsal (top view) shape of the epiblast generally was slightly oval with the short axis 70% as long as the long axis (Fig 1C). At later gastrulation stages the ratio is markedly shifted to greater ellipticity (data not shown). When sectioning embryos we noted progressive thickening of the epiblast to form a multi-layered lens-shaped mass of cells, reaching 35 to 45 μm in diameter at the thickest point. This occurred at stages when the epiblast was between 80 and 160 μm long (Fig 1D, see also Figs 2D, 2E, 3, 4, 5C, 6B, 7B and 8). The subsequent reduction in the thickness of the epiblast to between 16 and 30 μm reflects a transition to a 1–2 cell layered epithelium (Figs 1D, 2A, 2F, 3, 5G, 6I, 7E, and 9C). We shall refer to this epithelialized layered epiblast as embryonic ectoderm (EmE). The change in epiblast thickness from 150 μm occurred concomitantly with an overall change in the profile of the embryonic disk which first became flat, then progressively more convex-shaped, protruding out of the surrounding trophoblast (Figs 1E and 3). Once the EmE is of a maximal diameter exceeding 250 μm, a thickening can be observed at one end indicative of the formation of the primitive streak region (Figs 1G, 3, 5H and 9D). A dissolution of the basal layer of this region can be observed as well as the delamination of (mesendodermal) cells that are migrating between the EmE and hypoblast as well as inserting into the hypoblast (Fig 5H).

Interestingly, many of the thicker lens-shaped epiblasts (between 100 and 150 μm long) developed extracellular cavities (Fig 1E, examples in Figs 2A–2C, 5C and 8, S1 Fig). These transient cavities could be substantially enlarged in the most advanced of these embryos, with the dorsal (outer) cells stretched thinly over the cavities while the more ventral epiblast under the large cavities was already forming a 2-cell layered concave (protruding into the embryo) embryonic ectoderm (Fig 2A–2C). Importantly, the dorsal layer of cells covering the cavities is definitively of epiblast origin as opposed to being a remaining layer of polar trophoblast/Rauber’s layer cells. This was shown by staining for the epiblast marker *CRIPTO* (Fig 2A–2D). Thus we can conclude that these are intra-epiblast cavities and that a rupture of Rauber’s layer is unlikely to be involved in the transition from the lens-shaped epiblast to the flat/convex-dome shape seen at embryonic ectoderm stages.

The polar trophoblast overlying the epiblast, namely Rauber’s layer, disintegrates between E11 and 13 (Figs 2E, 2F, 6C, 7B and 8D). We defined a semi quantitative scale for its disappearance, where “1” represents an intact Rauber’s layer and “5”, no Rauber’s layer (Table 1). Using this classification to map the state of Rauber’s layer to epiblast length revealed a gradual demise of Rauber’s layer between 100 and 170 μm that could be approximated by a sigmoidal power function (Fig 1F). Below 100 μm epiblast size, Rauber’s layer had not started disintegrating while by 165 μm it was always gone. The starting point and completion of disintegration were variable, with intact membranes seen up to an epiblast size of 130 μm, whereas some embryos had already lost all or most of the layer by 145 μm (Fig 1F).

**Table 1:** Staging system used for classifying disappearance of Rauber’s layer (RL).

RL stage	Description
**1**	RL intact, no holes
**2**	RL starting to disintegrate, holes smaller than 10% of epiblast length
**3**	RL disintegrating
**4**	Remnant of RL, mainly along edges (10% of epiblast length)
**5**	RL gone, odd cell may still be seen

Morphologically, we observed changes in the hypoblast at two stages (Figs 1G, 3 and 7B). At epiblast sizes of 90 μm, the hypoblast cells underlying the epiblast became larger/more cuboidal than those backing the mural trophoblast. The epiblast-subjacent hypoblast is herein referred to as *visceral* hypoblast (VH) and that underlying the mural trophoblast as *parietal* hypoblast (PH), using the Greek-derived terminology used for rodent primitive endoderm. By epiblast sizes of 120 μm, a new population of visceral hypoblast cells arises that is taller/more columnar and often extends fine processes toward the epiblast (Figs 1G, 3, 5C, 8B and 8D; S2 Fig). These cells are asymmetrically localised, extending from the distal interior extremity of the concave shaped embryonic disc toward one (lateral) edge. Serial sections suggest this region to be circular or oblong as seen from a top (dorsal) view of the embryo (Fig 5A and 5B). As these cells express the anterior hypoblast marker *CER1* (discussed in more detail later), we term this distinct region the anterior visceral hypoblast (AVH).

### A staging system based on morphological and size criteria

By assembling all these criteria related to the variable of maximal epiblast length, we can now model the early developmental events into a detailed morphologically based staging system (Fig 3). We have numbered the five stages and termed them by their most characteristic embryological event. Post hatching, the hypoblast covers the inner (blastocoel) surface of the epiblast and mural trophoblast as a layer with widely spaced nuclei and cells containing a flat, thin cytoplasm. The 60 to 90 μm discoid epiblast, 1–2 cells thick, is fully covered by Rauber’s layer. This is stage 1 or Rauber’s Layer (RL) stage, seen in spherical blastocysts of 0.3 to 0.4 mm at E10-11. Over the next day, embryos continue growing to reach a slightly ovoid shape up to 1 mm in length (0.8 to 0.9 mm shorter axis). During this phase the epiblast has increased in size both laterally (90 to 120 μm) and in thickness. This stage 2 is termed the VH stage as the visceral hypoblast underneath the epiblast can be distinguished from the parietal hypoblast underlying the mural trophoblast by virtue of higher cell density and a more cuboidal cell shape. Rauber’s layer is beginning to disintegrate. The third stage, the AVH stage, is characterised by the further differentiation of the visceral hypoblast into an anterior specialised domain spreading from beneath the centre of the epiblast to one disc edge with more columnar cells extending processes toward the epiblast. This specialised visceral region will be referred to as the anterior visceral hypoblast, or AVH. Furthermore the multi-layered epiblast frequently exhibits extracellular cavities and has enlarged to a maximal disc diameter of 120–160 μm (if cavities persist, up to 180 μm). Rauber’s layer is generally broken up but not fully gone. Embryos exhibit these characteristics generally at age E12-13 and mural trophoblast proliferation is causing embryo extension mainly along one axis with embryos 0.9 to 1.4 mm long (width 0.8 to 0.9 mm). In the fourth, EmE-stage, the epiblast has “popped” out of the blastocoel cavity to protrude as a two-cell EmE layer from the surface plane of the mural trophoblast (S2 Fig). The still slightly oval embryonic ectodermal disc measures 150–260 μm across in embryos of up to 5 mm length. As these embryos are still mostly 1 to 1.5 mm in width, they start appearing “tubular”. Starting at E14 but usually only by E15, embryos more than 5 mm in length begin gastrulation with a slight thickening of the posterior embryonic ectoderm region indicating the formation of the primitive streak. Mesendoderm cells are seen migrating between the EmE and VH, whereas extraembryonic mesoderm cells are moving posteriorly between the mural trophoblast and parietal hypoblast. We defined this stage as the stage 5-early gastrulation (EG) stage.

### Gene expression from AVH to EG stages

To obtain a more mechanistic idea of the early embryonic events described above in terms of morphology, we examined the expression of developmental genes shown to be relevant for similar processes in the mouse. The embryonic disc region was cut from the rest of Stage 3 to 5 embryos and RNA isolated from the two regions. Stage 3-AVH discs contained Rauber’s layer, epiblast, AVH and VH while developmentally later discs no longer contained Rauber’s layer trophoblast. The non-disc fraction consisted of mural trophoblast (mTB) and parietal hypoblast.

We measured *FURIN* expression, as *FURIN* has previously been shown to be expressed in trophoblast in cattle [37] and is expressed in the mouse extraembryonic ectoderm [24], which, similar to Rauber’s layer, is a polar trophectoderm derivative. *FURIN* expression increased slightly with stage in the mTB fraction but was also seen in the stage 3 disc region at higher levels than in the mTB fraction. Levels were much lower in later stage discs (Fig 4A). The high levels at stage 3, when parts of RL are still present, suggests that *FURIN* may be expressed at higher levels in RL relative to the mTB.

We noted only background expression of *CER1* in the mTB/PH fractions at all stages but distinct expression in the AVH-containing stage 3 and 4 discs. With the start of gastrulation *CER1* levels doubled, presumably due to the combined expression in remaining AVH and nascent mesendoderm cells (Fig 4B).

Expression of the early mesoderm/prospective mesoderm marker *EOMES* was detected not only in stage 5-EG embryos, but also in earlier discs (Fig 4C). While all these results were quantitative, they gave insufficient indication of the spatial gene expression patterns, which we addressed next, using whole mount in situ hybridisation.

### Expression of genes involved in asymmetry establishment

A morphologically distinct region in the hypoblast (primitive endoderm), located to one side of the epiblast disc, is the first indication of asymmetry in the mammalian embryo. No expression of *CER1* was detected in stage 1 or 2 (RL/VH) embryos. At its onset in stage 3-AVH embryos, when expression is still very faint (Fig 5A), cattle *CER1* is already asymmetrically expressed in morphologically distinct hypoblast cells located in an oblong area stretching from the centre of the embryonic disc towards one margin (Fig 5B–5D). Later, *CER1* expression continues to track AVH cells until stage 4-EmE (Fig 5E and 5G).

*NODAL* expression was seen as early as stage 1-RL, located exclusively in all the epiblast (Fig 6B). Concomitant with the morphological distinction of the visceral and parietal hypoblast (Stage 2-VH), *NODAL* was additionally intensely expressed in the VH. At later stages it continued to be expressed in the visceral hypoblast with greater intensity in the AVH region (Fig 6E–6I).

Very recently, the T-box containing transcription factor Eomes has, similar to Nodal, been shown to be important in the development of the AVE [38]. Indeed, bovine *EOMES* was intensely expressed in the VH but not in the PH of stage 2-VH embryos (Fig 7C). Very weak *EOMES* expression was also seen in the epiblast. *EOMES* continues to be expressed at later stages all over the VH with no obvious anterior-posterior restriction (Fig 7D–7F).

Lastly, when examining *BMP4* expression, we found it to be expressed in the VH in a reciprocal pattern to that of *CER*, in that it was specifically *not* expressed in the AVH (Fig 8). Notably, expression also extended a couple of cell diameters into the circumferential parietal hypoblast (Fig 8A, 8E and 8G). This suggests that there may be functional differences within the parietal hypoblast.

### Gene expression in the trophoblast and a novel dorsal to ventral patterning of the early epiblast

After the initial homogenous expression of *NODAL* in the 1-RL stage epiblast, expression is shut down in a dorsal (outside) to ventral fashion resulting in a lack of *NODAL* transcription in the epiblast region that was originally covered by Rauber’s layer (Fig 6D). The NODAL cofactor *CRIPTO* shows no such restriction, remaining ubiquitously expressed in the epiblast at stage 3-AVH (Fig 2A–2D). We examined expression of the convertase FURIN at the cattle VH stage. Strong expression was seen in Rauber’s layer (Fig 2E). However, Rauber’s layer disappears at about the stage that *NODAL* expression is lost from the dorsal epiblast. If the FURIN-fuelled NODAL autocatalytic loop seen in the mouse [27] is also active in cattle, Rauber’s layer disappearance may thus be causal for the lack of *NODAL* maintenance in the dorsal region of the cattle epiblast.

Notably, *BMP4* is ubiquitously expressed in the epiblast at the AVH-stage and is not expressed in Rauber’s layer (Fig 8). Nor does Rauber’s layer express *EOMES* at any stage (Fig 7).

### Getting ready for gastrulation

By stage 4-EmE, *NODAL* is restricted to the posterior EmE region where the primitive streak will eventually form (Fig 6H–6M). *EOMES* is similarly expressed in the posterior EmE, well before gastrulation commences and mesoderm cells emanate from the streak region (Fig 7F). Interestingly the pan-mesoderm marker *BRACHYURY* is also already expressed at this pregastrulation stage (Fig 9A and 9C). *BRACHYURY* is initially detected in a posterior stripe covering the posterior-most 10–20% of the disc. Once cells delaminate from the thickened posterior EmE, *BRACHYURY* is still seen as a posterior stripe in dorsal views (Fig 9B), but does not yet label the first cells that gastrulate (Fig 9D). At later stages (beyond the here described 5-EG stages) *BRACHYURY* also labels the embryonic, but not extraembryonic mesoderm cells (to be published elsewhere).

The EmE expression of *CER1* commences later than *BRACHYURY*, at stage 5-EG, and never extends as far posterior and presumably marks the anterior portion of the forming primitive streak from which mesendoderm cells emanate (Fig 5G and 5H). Indeed *CER1* expression can be detected in cells that have inserted themselves into the hypoblast layer and thus are presumptive endodermal cells (Fig 5H). Concomitantly, expression is lost in the AVH (Fig 5F and 5H). This occurs together with the loss of a morphologically distinctive AVH population.

## Discussion

We present here a novel staging system for cattle embryos between hypoblast formation and the start of gastrulation. The system is based on developmental events during this time period and is substantiated by the expression of molecular markers characteristic of these events. Our staging represents a refinement of previously described morphologically-based staging systems for ruminants (cattle and sheep) in that the 2-VH to 4-EmE stages described here correlate to the single “Pre-streak stage 1” in these publications [22, 39]. A refinement in pregastrulation stages is necessary to provide a framework for the description of the important sequence of patterning events that take place during this period.

This study used only in vitro produced embryos which were transferred at the blastocyst stage to synchronised recipient animals. This ensures a more homogenous timing of staging compared to artificial insemination by avoiding the uncertainty in the timing of ovulation. However in vitro culture and transfer of several embryos into single recipients may cause changes in subsequent development that are not addressed further.

From a practical point of view our data indicates that a simple measurement of epiblast maximal diameter gives a rapid and reasonably good approximation for an embryo’s developmental stage during this time period. Conversely, if an embryo is staged according to epiblast length, but does not meet one or more of the attributes characteristic for that stage (such as size, hypoblast and epiblast differentiation and RL-state), this may indicate defective development and thus inferior quality or developmental capacity of that embryo. This is exemplified by the high correlation in epiblast size and embryo length. Embryo length at these stages measures the expansion/proliferation of the trophoblast, which makes up the outer surface that is measured. It can therefore be concluded that trophoblast and epiblast proliferation are largely synchronised until the start of gastrulation, and deviations could be indicative of defective development. Hence a chronological age-independent assessment of embryo quality can be achieved, allowing for example a better insight into the causes of the high levels of dairy cow embryo mortality.

### Comparing early morphological events in cattle to other mammals

We describe here two morphological events, namely AVH formation and cavity formation within the epiblast, both of which, to our knowledge, have not previously been described in ruminants. Cavity formation within the epiblast occurs predominantly during the 3-AVH stage. A similar event was recently reported for pigs [40]. We suspect that the formation of large cavities, presumably by coalescence of smaller ones within the epiblast results in tensional strain such that upon rupture of the thin dorsal layer, the ventral embryonic ectoderm can then either flatten or buldge out in a convex manner leading to the rapid change in shape seen in transverse views of the embryonic disc region. Whereas cavity formation in mice and human epiblast lead to the formation of a proamniotic cavity, in cattle and pigs cavity formation appears to be involved in the transition from the multi-layered mass of epiblast cells to the one to two cell layered flat epithelial-like embryonic ectoderm, which remains directly exposed to the maternal environment for several days. It has been postulated that the epithelialisation of the epiblast, achieved via different trajectories in birds/reptiles, monotremes, marsupials, and even among the different classes of eutherian mammals is a necessary requirement in amniotes [41].

The other event is the specialisation of part of the visceral hypoblast into what we term the AVH. The AVH is characterised morphologically by a thickening of the VH through a combination of cell shape change to a more columnar form and the formation of cellular processes extending towards the base of the epiblast. Genetically the AVH specifically expresses *CER1*, as does the mouse AVE [29, 42]. In mice the AVE has been shown to be required for the formation of the head (anterior) region. This is postulated to occur through its secretion of Lefty and Cer1, which upon diffusing into the adjacent epiblast inhibit the organiser-inducing factor Nodal, thereby restricting gastrulation to the prospective posterior parts of the embryo. The embryonic ectoderm overlying the AVE is thereby freed to assume an anterior character either by default or via further instructive interactions with the underlying AVE region [43]. The demonstration here that *CER1* is specifically expressed in the cattle AVH suggest an axis specification process analogous to that driven by the mouse AVE. The AVH thus is the first indication of anterior-posterior asymmetry in cattle embryos.

A major question remains as to how the AVH arises in the first place. In mice the AVE is preceded by the distal visceral endoderm (DVE), a localised thickening of the VE at the distal tip of this cup shaped layer. The DVE expresses similar genes to the AVE [44]. If we flattened out the cup-shaped mouse epiblast and VE, the DVE would lie in the centre of the resultant disc. It thus does not mark an anterior-posterior axis. This axis is determined shortly after, via the unidirectional movement of DVE cells towards one edge of the disc. Notably, these migrating DVE cells do not actually give rise to the AVE. The AVE forms from VE cells replacing, and then following in the wake of, the migrating DVE population [45]. When the DVE is genetically ablated, the AVE forms, but fails to migrate anteriorly [45]. The role of the mouse DVE is therefore to guide the anterior migration of AVE cells, but it is unclear how this unidirectional motion is achieved.

Rabbits are more closely related to rodents (84 mya; million years ago) than either are to ruminants (94 mya) [46]. In rabbits the AVE-equivalent region, termed the AMC (anterior marginal crescent), is morphologically first visible in an asymmetric fashion on one end of the flat embryonic disc as columnar hypoblast cells expressing *CER1* [16, 17]. Before the appearance of morphologically distinct columnar hypoblast cells, a few centrally located *CER1*-positive cells were seen, suggesting the existence of a DVE-like region in this species as well [17].

We did not detect a structure equivalent to the mouse DVE, either because it does not exist in cattle or because such a structure is located only very transiently in the centre of the disc. We suspect the former possibility as at the very earliest faint onset of expression, *CER1* was already asymmetrically expressed. In pigs, which are relatively closely related to cattle (61 mya), no centrally located DVE-like hypoblast structure was detected either. Morphologically distinct hypoblast (termed AHB in pigs) was already asymmetrically located at the earliest stages examined (130 and ca 100 μm epiblast in references [20, 40] respectively).

**Fig 10:**
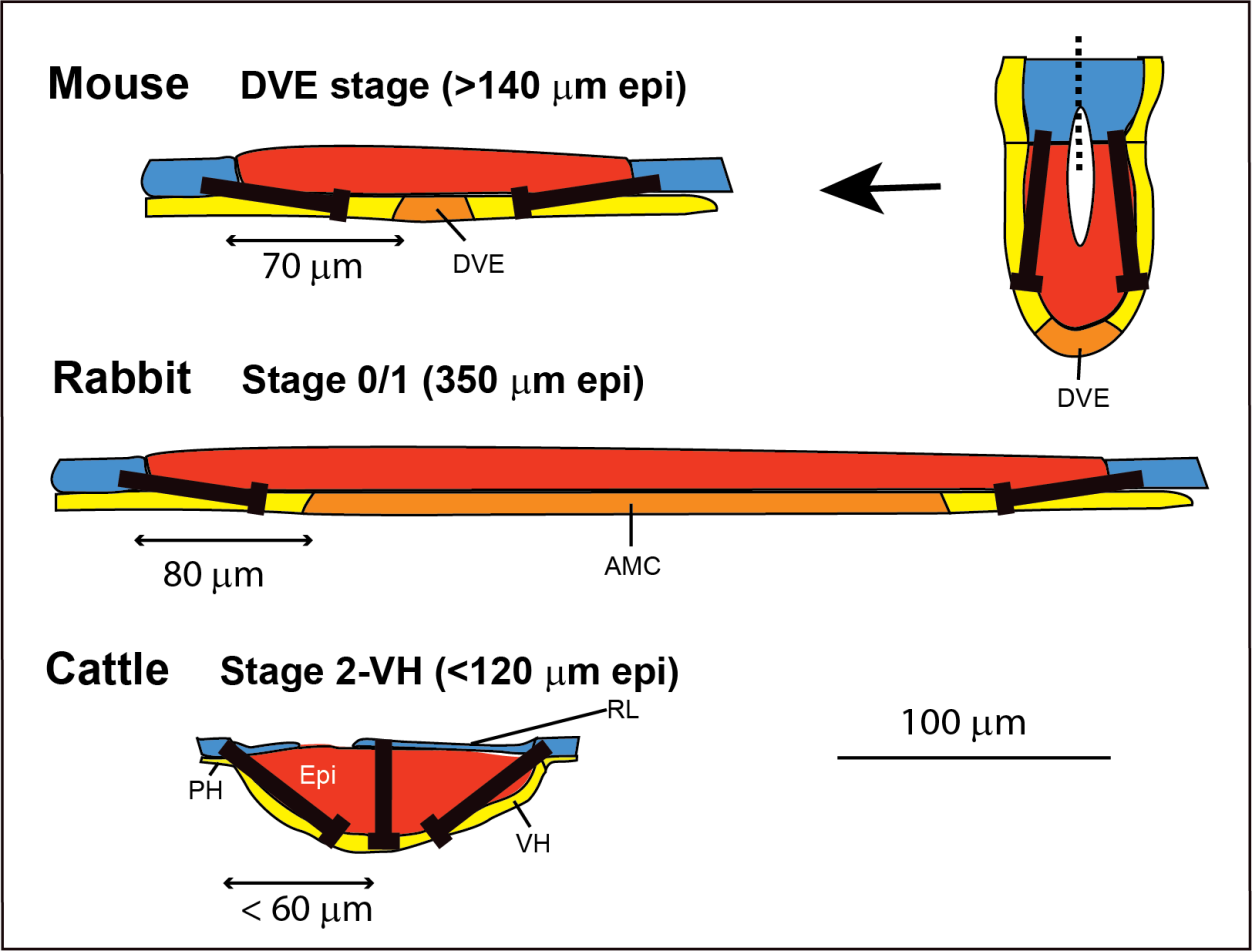
Comparison across several species of establishment of hypoblast signalling centre. In mice, negative signals (black arrows; BMP) from the trophoblast (in blue) are believed to inhibit the establishment of a distal hypoblast signalling centre until the epiblast (in red) has proliferated sufficiently to remove some of the visceral hypoblast (in yellow; in mice termed the visceral primitive endoderm, or VE) from this influence allowing the DVE (orange) to form at distances greater than 60 μm. A conceptual flattening out of the mouse egg cylinder is shown. In rabbits, CER1, marking the initially centrally and symmetrically located DVE equivalent, termed the AMC, again forms at a distance from the trophoblast margin. In cattle, persistence of the polar trophoblast (Rauber’s layer) and a more lens-like transverse shape of the epiblast infer that the VH remains under the influence of putative inhibitory signals, precluding a DVE-like precursor population of the AVH.

**Fig 11:**
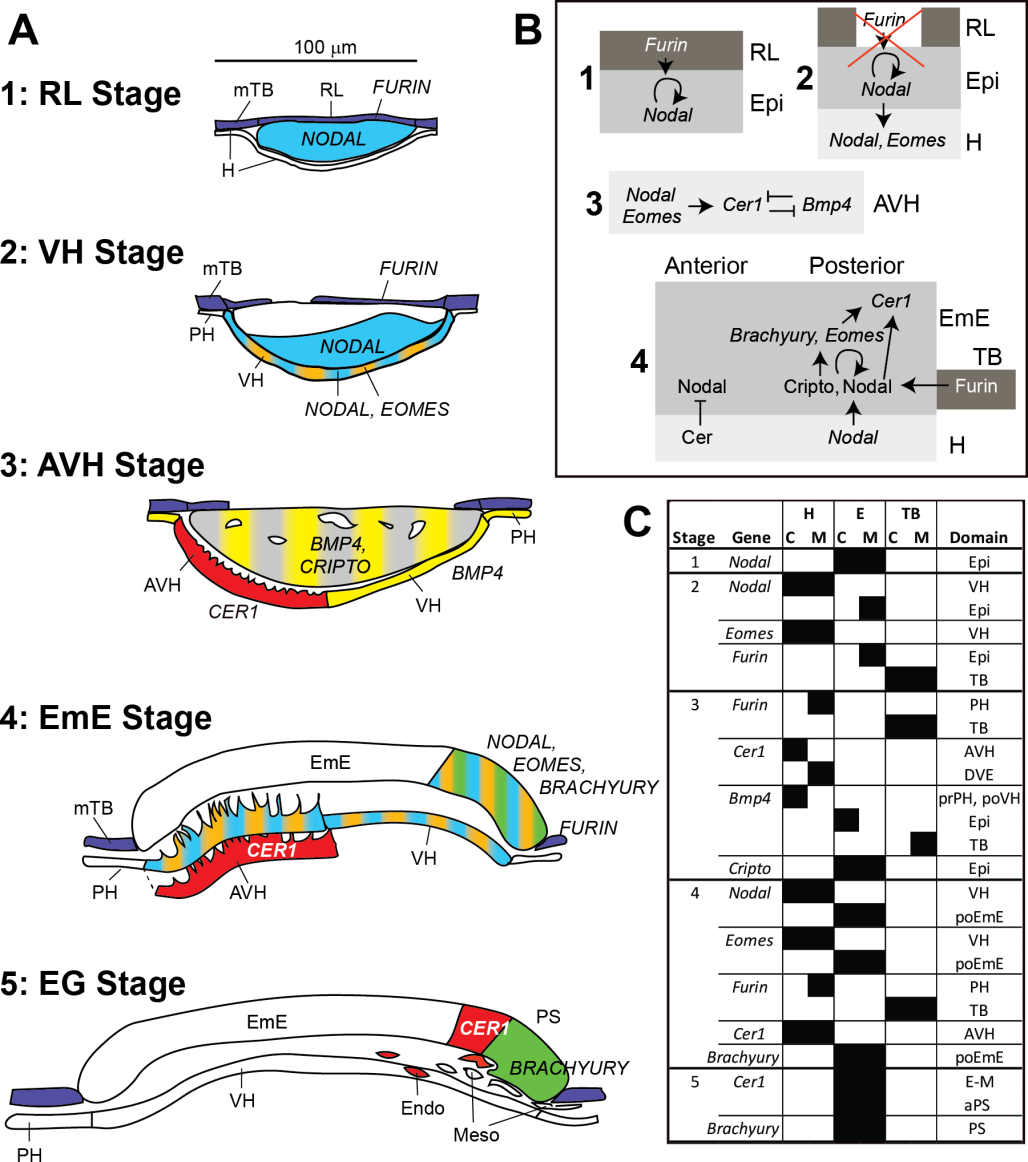
Summary of gene expression patterns between stages 1-RL and 5-EG, potential interactions and comparison to mouse embryos. **A.** Expression of genes are shown at the stages analysed and colour coded: *FURIN* dark blue, *NODAL* light blue, *EOMES* orange, *CRIPTO* grey, *BMP4* yellow, *CER1* red and *BRACHYURY* green. **B.** Gene/protein regulatory interactions as described in mouse embryos are depicted in shaded boxes representing trophoblast (dark), epiblast/EmE (mid) and hypoblast (light). References are listed in the discussion. **C.** Comparison of gene expression domains in mouse (M) and cattle (C) embryos at cattle stages 1–5. The mouse equivalent stages are described in Table 2. *aPS*, anterior primitive streak; “*Epi”* refers to expression in all of the epiblast; *prPH*, proximal parietal hypoblast/endoderm; *po*, posterior.

The reason for the postulated absence of a DVE-like structure in cattle and pigs and its presence in mice and rabbits may be understood if one considers the shapes and sizes of the various embryos (Table 2 and Fig 10). In mice, as the epiblast grows, it eventually physically distances the most distally located VE from the inhibitory influence of the polar trophoblast (the ExE), allowing the up-regulation of *Cer1* and other DVE markers [47, 48]. This occurs when the distance from the epiblast-trophoblast border exceeds 70 μm [47], or, in a flat disc scenario, when the disc diameter exceeds 140 μm. Rabbit embryos have an unusually large embryonic disc of about 500 μm and have lost the polar trophoblast (Rauber’s layer) at stage 1 when the AMC (AVH, AVE) is first morphologically visible [16]. The central *CER1* expression in rabbits is detected just before stage 1 but after Rauber’s layer disappearance and may thus, as in the mouse, be due to this hypoblast region having escaped the influence of an inhibitory signal from the circumferential trophoblast. In support of this idea, the initial central *CER1* domain in the rabbit only begins about 80 μm from the disc edge (for in situ evidence see Fig 3A in [17]). In cattle (and pig) embryos, the much smaller epiblast disc size (maximal edge to centre distance of 60 μm at late stage 2-VH), as well as the persistence of much of RL up to stage 3-AVH, precludes the escape of any VH from an inhibitory trophoblast-derived signal (Fig 11). Thus AVH formation may rely on mechanisms that do not involve an initially centrally located migratory “precursor” cell population.

**Table 2:** Morphological comparison of stage-matched cattle and mouse embryos.

Stage	Species	Alt	Age	Tissues	Epi (μm)^a^
**1-RL**	cattle		10.5	pTB,Epi,H	75
	mouse		5.0	ExE,Epi, H	80
**2-VH**	cattle		11.5	pTB-holes,Epi,VH-PH	105
	mouse	preDVE	5.3	ExE,Epi-cavity, VE-ExVE/PE	105
**3-AVH**	cattle		12.5	pTB-disapp,Epi-thick-cavities,AVH	150
	mouse	DVE	5.5	ExE,Epi,DVE	160
	rabbit	st-0/1	5.9	pTB-holes,Epi,DVH	340
**4-EmE**	cattle		13.5	mTB,EmE,AVH	200
	mouse	AVE	6.0	ExE,EmE,AVE	200
	rabbit	st-1	6.0	pTB-disapp,Epi,AMC	500
**5-EG**	cattle		14.5	mTB,EmE,PS, Endomes,VH	300
	mouse	EG	6.5	ExE,EmE,PS,Endomes,AVE	300

^a^ For mouse embryos proximal-distal length has been doubled to reflect a “flattening out” of the cup shaped epiblast; references for epiblast diameters: [17, 47, 49].

One AVH-induction mechanism potentially could involve the polar trophoblast (Rauber’s layer). In the mouse, the extraembryonic ectoderm trophoblastic tissue (ExE) is derived from the polar TE and can thus be considered homologous to the RL. This ExE has been postulated to be the source of a DVE-inhibitory Bmp signal [47, 50]. Secondly, the ExE secretes convertases into the epiblast to activate Nodal signalling via an autocatalytic loop [24, 27]. We have shown here in cattle expression of *NODAL* in the epiblast and its convertase *FURIN* in the RL. However, in cattle RL disappears from stage 2-VH onwards. This occurs in an irregular fashion with embryos displaying holes of varying sizes and number in this layer. Such irregularities may be the initial trigger for subtle differences/gradients in NODAL signals in the underlying epi- and hypoblast layers that could subsequently be amplified via reciprocal feedback loops leading to the asymmetrical establishment of the AVH signalling centre. We are currently testing this possibility.

### Conservation of, and variation in, the molecular machinery underlying early mammalian patterning

We have examined here, in various stages of cattle embryos, a number of genes whose homologues have been shown to be critical for embryonic patterning in the mouse. This data is summarised in Fig 11A. Comparing the expression domains of the various genes with those seen in well-documented mouse embryos at equivalent stages (Fig 11C) reveals a high concordance of expression at the later stages of 4-EmE and 5-EG, but more variability during stages 2-VH and 3-AVH. The greater earlier variation corresponds to the variation in early morphological events seen in these two species, namely (i) RL disintegration versus ExE proliferation, and (ii), direct AVH formation versus a DVE-AVE sequence. Particularly noteworthy are a), the early loss of *NODAL* in cattle epiblast, b) the radically different expression of *BMP4* and c), the more restricted expression of the convertase *FURIN* in cattle embryos (Fig 11C).

In the earliest (1-RL) cattle embryos described here, there is no evidence for morphological differentiation or molecular patterning within the trophoblast, epiblast and hypoblast lineages. *NODAL* is expressed all over the epiblast, but not yet in the hypoblast, in concordance with early peri-implantation E4.5–5 mouse embryos [47, 51].

In mice, the Nodal activity in the epiblast is required for the underlying primitive endoderm/hypoblast to differentiate into VE in pre-DVE-stage E5-5.3 embryos [47, 48]. During this process Nodal protein diffuses into the hypoblast to switch on *Nodal* [27] and *Eomes* expression [38]. These hypoblast inductive events appear to be fully conserved in cattle embryos, as substantiated by *NODAL* and *EOMES* expression in the morphologically distinct VH cell population seen at stage 2-VH. However gene expression in the epiblast differs. In mice both *Nodal* and *Furin* are expressed all over the epiblast [47, 51], whereas in cattle *FURIN* is excluded from the epiblast and *NODAL* expression is progressively lost from outside to inside. The loss of *NODAL* transcriptional maintenance in cattle epiblast may be related to the loss of RL and thus of the last available (non-epiblast) source of FURIN (Fig 11B). The resultant absence of FURIN would lead to suboptimal NODAL preprotein activation and abrogation of the *NODAL* autoregulatory loop. What effect could the early shutdown of *NODAL* in the cattle epiblast have? Nodal expression in the mouse epiblast fulfils two probably interrelated functions – epiblast cell proliferation and DVE formation [47]. As epiblast proliferation removes the distal hypoblast from the negative influence of the ExE, it indirectly supports DVE formation. Thus in cattle, we would predict reduced epiblast proliferation and hence the absence of a DVH. Indeed as discussed previously, we see no cattle DVH and whereas the mouse epiblast radius increases by over 100% per day (e.g. 50 μm to over 100 μm between E5.5 and 6.5 [49]), the cattle epiblast increases only by 35% a day (from Fig 1A; d/dx of e^0.30x^). Lastly, cattle epiblast expression of *BMP4* is not seen in mice [25]. Indeed, in mice Nodal appears to repress *Bmp4* transcription in the epiblast [52], whereas it is required for Bmp4 expression in the adjacent ExE [24]. The early shutdown of *NODAL* in the cattle epiblast may thus lead to a permissive environment explaining the widespread expression of *BMP4* in this tissue at stage 3-AVH.

In mice, Nodal and Eomes have been shown to be required for *Cer1* transcription in the AVE as well as for AVE migration [38, 51]. As expected from the conserved pattern of *NODAL* and *EOMES* expression in the cattle and mouse VH, *CER1* expression is conserved, being exclusive to the morphologically distinct cattle AVH (from stage 3-AVH on), as previously discussed. Unpredictably though, the AVH can also be defined by the absence of *BMP4* expression in cattle. In mouse embryos *Bmp4* is exclusively expressed in the ExE [25] and has been proposed to be the inhibitory trophoblast signal that diffuses into the VE to restrict the formation of the DVE to the distal-most region. Before *Cer1* is expressed in the mouse DVE/AVE, Bmp signalling (via Smad1) is extinguished [48]. This exclusion of Bmp signalling from the mouse DVE is paralleled by the absence of *BMP4* transcription specifically in the AVH of cattle embryos. Thus a similar end result (absence of Smad1 activity in the hypoblast anterior signalling centre) may be achieved via different mechanisms in these two species.

Interestingly, expression of *BMP4* in the VH extended a few cells beyond the boundary of the embryonic disc but was not seen in mural trophoblast or in what remained of Rauber’s layer. In pig embryos of 140 μm epiblast diameter, a similar ring of expression extending beyond the disc was noted, though it was not determined which layer (TB or HB) exhibited this staining [19]. The function, if any, of these cells and whether these cells are analogous to a mouse embryonic population such as the ExE or extraembryonic VE, remains to be established.

The onset of *NODAL* in the posterior EmE at 4-EmE stage leads to a pattern that is spatially equivalent to that in mice where *Nodal* transcription is never switched off completely in the epiblast, but is progressively restricted to the posterior EmE quadrant. It will be interesting to determine whether this posterior *NODAL* expression/maintenance is driven, as in the mouse [24], by an autoregulatory loop, fuelled by FURIN from the posterior mural trophoblast/ExE and restrained anteriorly by NODAL inhibitors such as CER1 emanating from the AVH. The observed expression of *FURIN* in mural posterior TB and *CER1* in the AVH are compatible with such a mechanism in cattle, whereas our demonstration of expression of the NODAL cofactor *CRIPTO* in the EmE fulfils a further prerequisite for NODAL signalling activity.

In mice, Nodal, in the posterior ectoderm is required for the activation of *Eomes*, *Brachyury* and—together with Eomes—for the activation of *Cer1* [27, 38, 47]. This cascade, leading to gastrulation and mesoderm formation, appears to be conserved in cattle with *EOMES* and *BRACHYURY* expression commencing at stage 4-EmE, followed by *CER1* in the more anterior part of the forming primitive streak at stage 5-EG. As in the mouse [30, 31], *BRACHYURY* and *EOMES* expression precede the appearance of the first mesodermal cells, i.e. the start of gastrulation. In sheep, onset of *EOMES* has also been reported to occur concurrently with *BRA* but only after mesoderm formation [53]. This most likely is not a species difference, but rather reflects lower hybridisation sensitivity in that paper attributable to the use of a mouse probe for *Eomes* and a short (330 bp) probe for *BRACHYURY*.

*Eomes* and *Brachyury* are also expressed prior to gastrulation in the mouse ExE [26]. This ExE-specific trophoblast expression domain, similarly to that of *Bmp4* (discussed earlier) and *Elf5* [54], is not conserved in cattle, suggesting the absence in cattle of an ExE-homologous trophoblast domain. Such a homologous domain would have been expected—from shape considerations—to lie circumferentially around the embryonic disc.

In summary, we conclude that while the onset of gastrulation appears to be well conserved in terms of gene expression in mammals as diverse as cattle and mice, the mechanisms of asymmetry establishment, which relies on extraembryonic tissues such as the hypoblast and trophoblast, is more difficult to reconcile both in gene expression and morphological terms.

## Supporting Information

S1 Fig Sections showing intra-epiblast cavitation. Haematoxylin and eosin stained sections of A., B., stage 3-AVH and C., stage 2-RL embryos with cavities within the epiblast. Bars represent 100 μm.
(TIF)

S2 Fig Sections of Stage 4-EmE embryo stained for cytoplasmic membranes and nuclei. A. Cross section of *CER1* stained EmE-stage embryo. B. Same section showing hypoblast cellular processes extending toward EmE as revealed by phalloidin staining of actin filaments. C. Same section with nuclei visualised via DAPI staining. The EmE is seen to be 1–2 cell layers thick. D. Panels B and C are merged. Scale bar 100 μm. AVH, anterior VH; EmE, embryonic ectoderm.
(TIF)

S3 Fig Expression levels of housekeeping genes. Samples as for Fig 4.
(TIF)
