# Supplementary figures and images for: Morphological and Gene Expression Changes in Cattle Embryos from Hatched Blastocyst to Early Gastrulation Stages after Transfer of In Vitro Produced Embryos
Source: PLoS One. 2015 Jun 15;10(6):e0129787. doi: 10.1371/journal.pone.0129787 (PMC4468082; doi:10.1371/journal.pone.0129787)

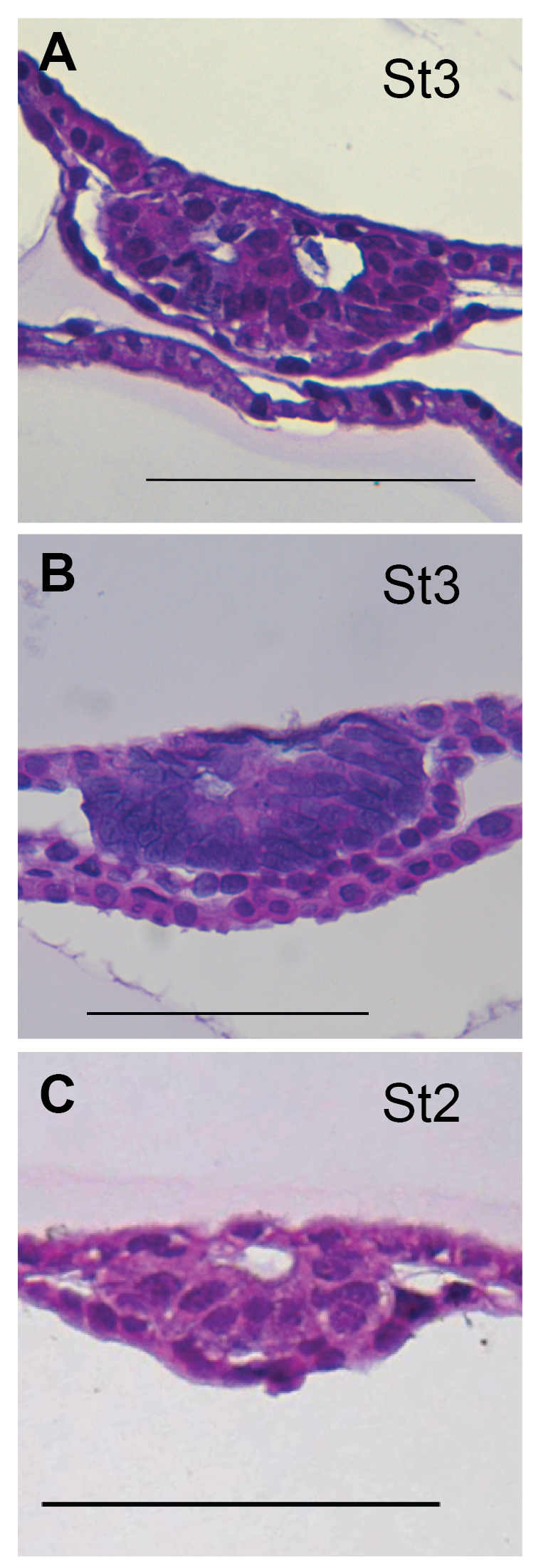

Supplement: S1 Fig — Haematoxylin and eosin stained sections of A., B., stage 3-AVH and C., stage 2-RL embryos with cavities within the epiblast. Bars represent 100 μm. (TIF) [file pone.0129787.s001.tif]

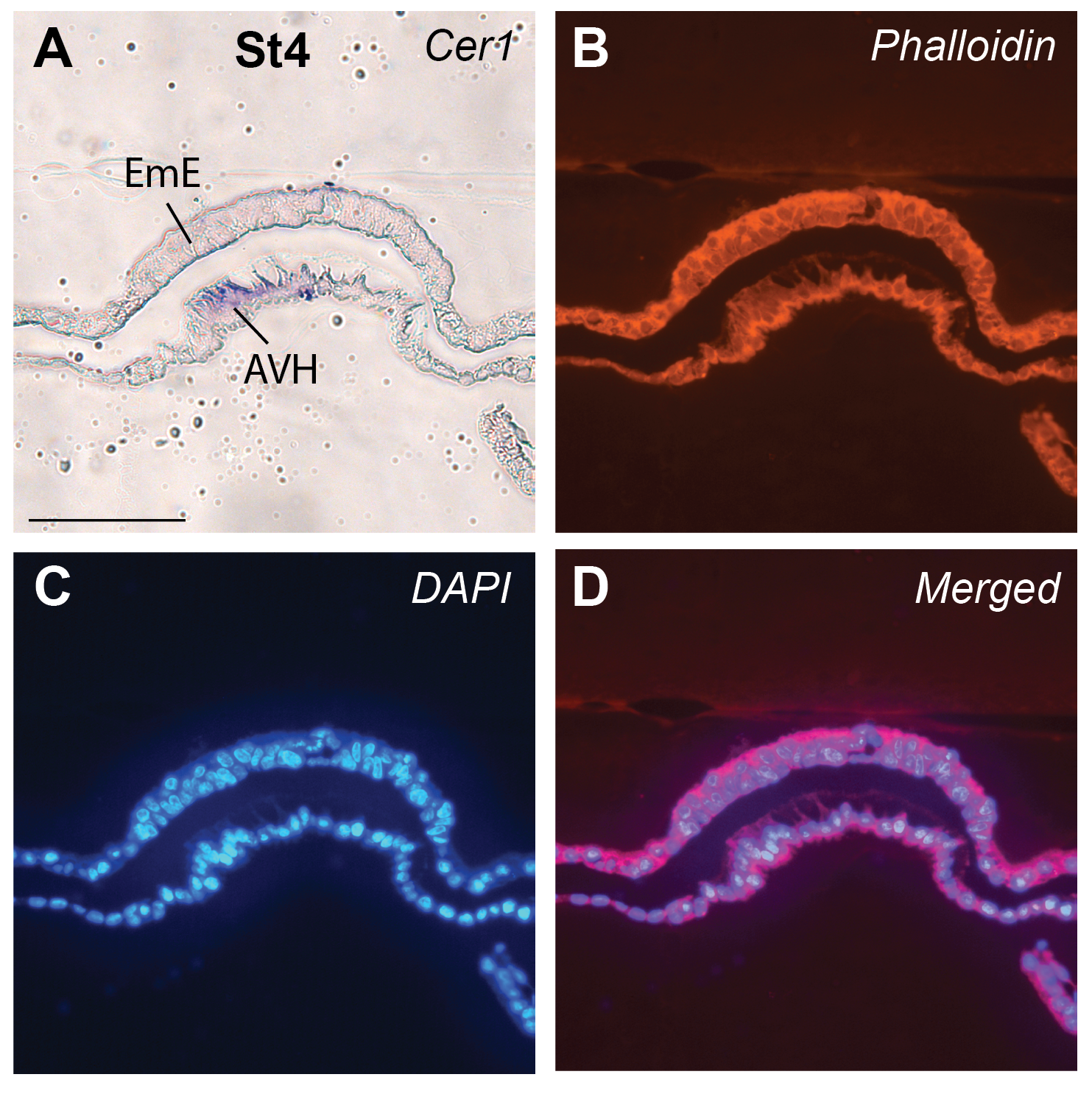

Supplement: S2 Fig — A. Cross section of CER1 stained EmE-stage embryo. B. Same section showing hypoblast cellular processes extending toward EmE as revealed by phalloidin staining of actin filaments. C. Same section with nuclei visualised via DAPI staining. The EmE is seen to be 1–2 cell layers thick. D. Panels B and C are merged. Scale bar 100 μm. AVH, anterior VH; EmE, embryonic ectoderm. (TIF) [file pone.0129787.s002.tif]

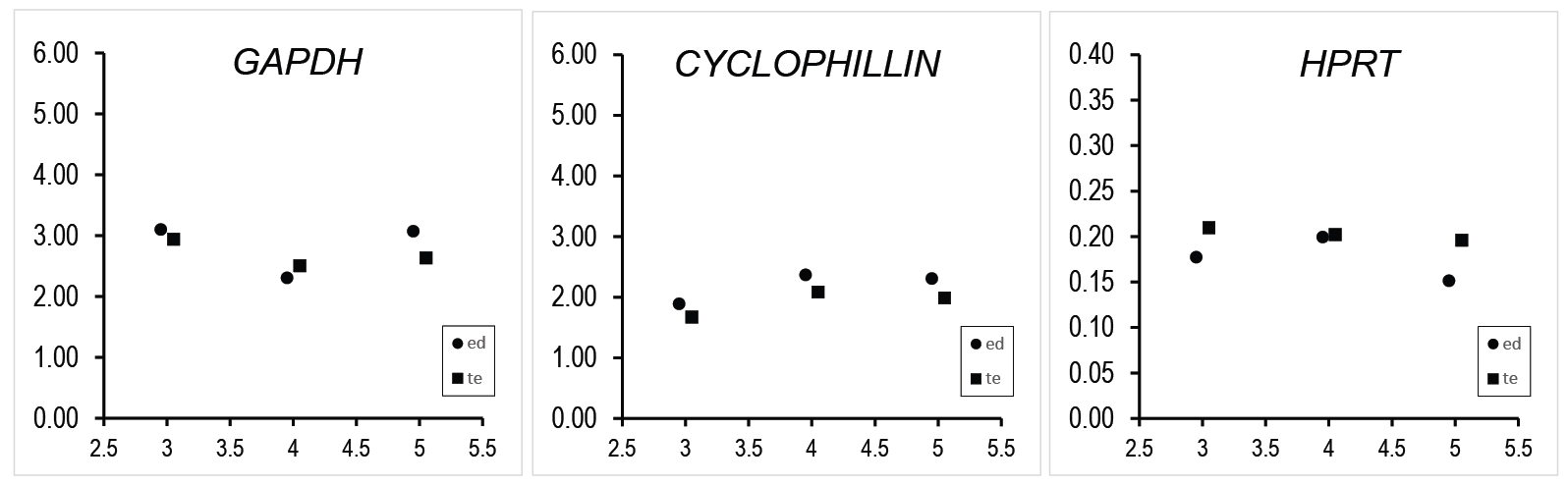

Supplement: S3 Fig — Samples as for Fig 4. (TIF) [file pone.0129787.s003.tif]
